# The Beginning of HCN Polymerization: Iminoacetonitrile Formation and Its Implications in Astrochemical Environments

**DOI:** 10.1021/acsearthspacechem.1c00195

**Published:** 2021-07-29

**Authors:** Hilda Sandström, Martin Rahm

**Affiliations:** Department of Chemistry and Chemical Engineering, Chalmers University of Technology, Gothenburg SE-412 96, Sweden

**Keywords:** prebiotic chemistry, C-cyanomethanimine, Titan, steered ab initio molecular dynamics, metadynamics

## Abstract

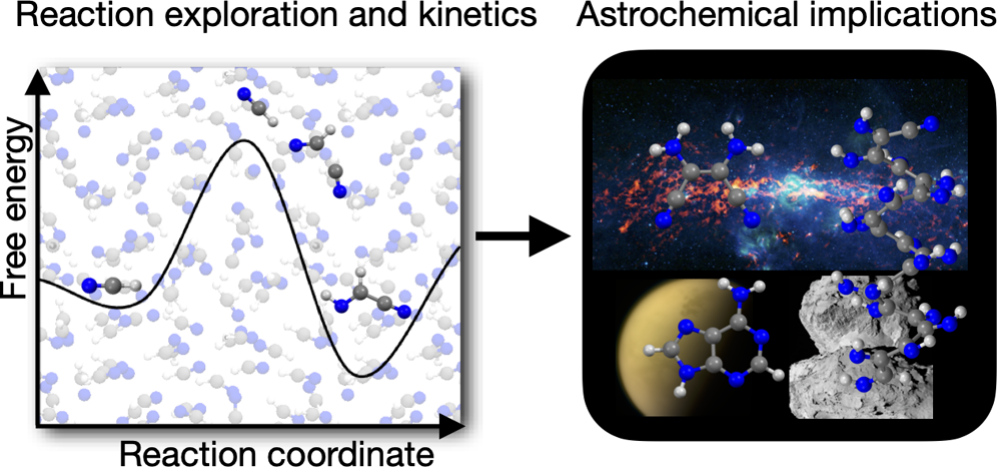

Hydrogen cyanide (HCN) is known to react with complex organic materials and is a key reagent in the formation of various prebiotic building blocks, including amino acids and nucleobases. Here, we explore the possible first step in several such processes, the dimerization of HCN into iminoacetonitrile. Our study combines steered ab initio molecular dynamics and quantum chemistry to evaluate the kinetics and thermodynamics of base-catalyzed dimerization of HCN in the liquid state. Simulations predict a formation mechanism of iminoacetonitrile that is consistent with experimentally observed time scales for HCN polymerization, suggesting that HCN dimerization may be the rate-determining step in the assembly of more complex reaction products. The predicted kinetics permits for iminoacetonitrile formation in a host of astrochemical environments, including on the early Earth, on periodically heated subsurfaces of comets, and following heating events on colder bodies, such as Saturn’s moon Titan.

## Introduction

**Figure 1:**
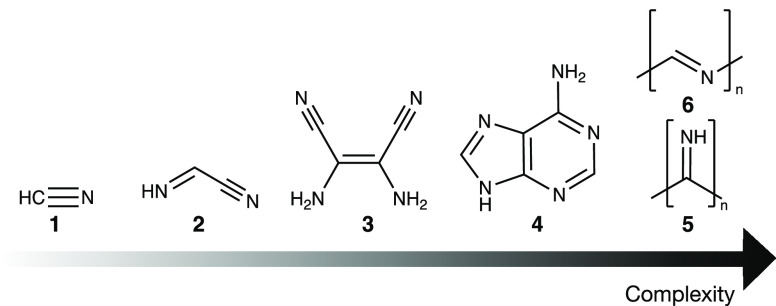
Selection of compounds proposed to form in HCN reaction mixtures. This work explores how HCN **1** can react to form iminoacetonitrile **2**. Iminoacetonitrile is a suspected key intermediate in the formation of more complex molecules, such as the HCN tetramer diaminomaleonitrile **3**,^17^ biologically relevant molecules such as adenine **4**^1^, and various polymers (e.g., **5**, **6**).^18,19^ Iminoacetonitrile can also be referred to as C-cyanomethanimine.

In this work, we use steered ab initio molecular dynamics to unveil the reaction mechanism for base-catalyzed formation of iminoacetonitrile (Figure 1, compound **2**), a suspected key prebiotic reaction intermediate, in liquid hydrogen cyanide (HCN).^1,2^ HCN is one of the most ubiquitous small molecules in the universe, having been observed in the interstellar medium,^3^ in the coma of several comets,^4^ in the atmosphere of the giant planets,^5,6^ on Pluto,^7^ as well as on Saturn’s moon Titan.^8^ Because of its high energy content and reactivity, HCN is a potential driving force for chemistry in various astrochemical environments.^9−11^ For example, both the Voyager Flyby and the Cassini mission to Titan have detected clouds made of HCN-based aerosols that can be expected to contribute to concentrated deposits on the considerably colder surface.^10^ Observations and modeling suggest that HCN can react to form various complex organic materials on Titan despite the low temperature.^12,13^ While there are large uncertainties regarding the nature of early Earth’s atmosphere, HCN is expected to have also been formed there as a product of reactions between nitrogen and methane following solar UV radiation, superflares, shockwaves, and discharge chemistry.^14−16^

**Figure 2:**
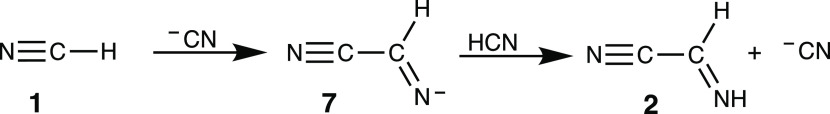
Schematic representation of the base-catalyzed iminoacetonitrile formation. Nucleophilic attack by a cyanide anion is followed by proton transfer.^1,2^

A long-standing hypothesis first put forth by Orò,^1^ Völker,^20^ Ferris, and colleagues^2^ posits that iminoacetonitrile, the most stable HCN dimer,^21^ is the first intermediate in the base-catalyzed polymerization of HCN in the liquid phase (Figure 2). The potential importance of this molecule has since been pointed out by many (e.g., refs (19, 22)) and it has been proposed as an early intermediate in the HCN-based synthesis of various biologically relevant molecules, such as purines, pyrimidines, pterins, and amino acids.^1,23−25^ HCN-derived polymers are also of interest as functional materials, e.g., in catalysis or as adhesives and coatings for biomedical applications.^13,22^ Given the presumed key role of iminoacetonitrile in prebiotic chemistry, it is important to answer the questions: where can this molecule form and how?

The formation routes to iminoacetonitrile can be expected to depend greatly on the chemical and physical environment. Iminoacetonitrile has recently been detected in the interstellar molecular cloud Sagittarius B2, using the Green Bank Telescope, and in the molecular cloud G + 0.693, using the Institute for Radio Astronomy in the Millimeter Range (IRAM) 30 m telescope.^26,27^ Several neutral,^28−30^ radical, anionic,^31^ and cationic^28^ reaction mechanisms have been explored computationally, in efforts to describe the formation of iminoacetonitrile from HCN in the gas phase. However, such mechanisms appear to correspond to either prohibitively high activation barriers^28−31^ or unfavorable thermodynamics^28^ to allow for meaningful (thermal) reaction rates in the interstellar medium. Computational studies of HCN dimerization on ice grains have predicted similar unfavorable kinetics.^32^ A reaction network study suggests that the cyanide radical and methylene imine might be important precursors to iminoacetonitrile in molecular clouds.^33^

In this work, we focus on explaining the formation of iminoacetonitrile in neat liquid HCN. HCN polymerization reactions in polar solutions are known to be base-catalyzed and to proceed in a wide range of temperatures (195–373 K)^34,35^ at concentrations ranging from 0.01 M to pure HCN (26.2 M).^17,18^ However, while it has been hypothesized that iminoacetonitrile is required for the formation of many larger molecules,^1,19,36^ the compound has never been directly observed during HCN polymerization experiments. Iminoacetonitrile has been generated by pyrolysis of cyanoformamide salts and has been well characterized in the gas phase and in argon matrices by vibrational spectroscopy, mass spectrometry, and UV photoelectron spectroscopy.^37−39^ A ^1^H and ^13^C nuclear magnetic resonance spectroscopy study has also shown that iminoacetonitrile polymerizes rapidly above 233 K.^38^ Ferris and colleagues have shown that *N*-alkyliminoacetonitriles in aqueous cyanide solutions react to form maleonitrile derivatives, which are, in turn, likely polymer intermediates.^36^

To the best of our knowledge, there has only been one previous theoretical study of base-catalyzed iminoacetonitrile formation in polar solution by Kikuchi et al.^40^ That study relied on an implicit solvent model for water and predicted a reaction barrier (28.7 kcal/mol), which is markedly lower than the corresponding uncatalyzed pathway (103 kcal/mol^40^), but prohibitively large for reactions at ambient and lower temperatures.

## Results and Discussion

Our study of this condensed phase chemistry relies on molecular dynamics simulations. The use of dynamics simulations allows for the explicit consideration and sampling of the solvent environment and is important for three reasons: first, HCN is an exceptionally polar molecule. The dielectric constant of liquid HCN is 144.8 at 278 K,^41^ a factor of 1.7 larger than for water at the same temperature (85.8).^42^ Moreover, HCN is capable of donating and accepting strong directional hydrogen bonds. These properties of HCN share striking similarities with those of water and are suggestive of an unexplored potential for self-catalysis. Finally, the considered reaction mechanism involves charged species that interact both strongly and directionally with the environment.

### Reaction Exploration in Liquid HCN

We here rely on density functional theory (DFT) metadynamics simulations to identify viable formation routes of iminoacetonitrile from HCN while minimizing bias with regard to mechanism or solvent role. Our metadynamics approach relies on *path-collective variables* to define reaction coordinates. These variables are unbiased in the sense that they are only based on aspects of the coordination pattern of reactants and products. In our exploration, the reaction path is defined by an interpolation between the reactant and the product states. We use two path-collective variables: the *s* coordinate, which describes a structure’s position along the reaction path, and the *z* coordinate, which describes how distant a given structure is from the path.^43^ The simulation temperature of 278 K was chosen to be in the middle of the liquid range of HCN, which is close to that reported in recent studies on HCN polymerization.^18^

**Figure 3:**
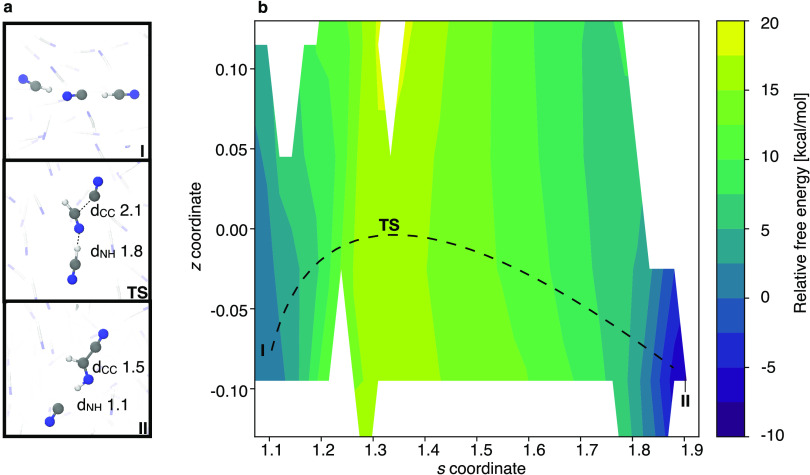
Beginning of HCN polymerization. Formation of E-iminoacetonitrile as predicted by a metadynamics simulation of liquid HCN in the presence of a cyanide anion. Panel a. The identified mechanism is concerted and involves carbon–carbon bond formation occurring simultaneously with a proton transfer between two HCN molecules. Distances are provided in Ångström. Panel b. The free-energy landscape of iminoacetonitrile formation in path-collective variable space. **I** represents the position of the reactant complex, **TS** represents the position of the transition state, and **II** represents the product complex. The white regions correspond to areas of the path-collective variable space that were not sufficiently sampled during the subsequent umbrella sampling simulations.

The reaction pathway observed in our simulations (Figure 3, panel a) resembles a concerted version of the mechanism initially proposed by Orò.^1^ The reaction proceeds through the formation of a carbon–carbon bond (d_CC_) between HCN and a cyanide anion that occurs concurrently with a proton transfer (d_NH_) from a second HCN, which, in turn, regenerates the catalyzing base. Representative snapshots from our simulations displaying geometries of reactant **I**, transition state **TS**, and product **II** are shown in Figure 3a. The **TS** has been identified through committor analysis, as the structure from which multiple simulation trajectories reach reactant and product basins with equal probability (see Methods section).

Solvent participation remains distinct along the entire reaction pathway, and at minimum two hydrogen-bonded HCN molecules actively partake. This is in line with previous simulations of liquid HCN, which have predicted HCN, on average, to have seven molecules as closest neighbors.^44^ The *s* coordinate allows us to assign the transition state as early (see the Methods section for details), in agreement with the Hammond postulate. The lowest-energy reaction profile for the observed formation of iminoacetonitrile was retrieved with umbrella sampling. Structures **I**, **TS**, and **II** are indicated in path-collective variable space in the resulting free-energy landscape shown in Figure 3b.

### Kinetics and Thermodynamics of Iminoacetonitrile Formation

Our simulations predict a relative Gibbs energy of −7.1 ± 0.8 kcal/mol for the overall reaction, suggesting that the mechanism is thermodynamically allowed (see Supporting Information Figure 3). The uncertainty range here refers to the error due to limited sampling during simulations (see the Methods section). Our simulations (viz. Figure 3a) predict the formation of the E-form of iminoacetonitrile. The other possible conformer, Z-iminoacetonitrile, has previously been calculated to be lower in energy in vacuum.^38,45,46^ However, in this study, the context is different: we consider iminoacetonitrile in a strongly interacting environment, and our best estimate puts the E-conformation at ∼1 kcal/mol below the Z-conformation at 278 K (Supporting Information Figure 5). The Gibbs energy difference between the two structures is small and within the accuracy of the methods we use. Consequently, the specific conformation of iminoacetonitrile does not affect our conclusion on the reaction mechanism nor its kinetics.

The Gibbs energy activation barrier, which corresponds to the TS structure in Figure 3, computes as 15.5 ± 1.2 kcal/mol from our simulations. However, there is a caveat: because of limitations in accuracy of practically feasible electronic structure methods, chemical rate estimation from ab initio molecular dynamics is a well-known challenge (see, e.g., ref (47)). The DFT method we use is a necessary compromise of quality and feasibility and one that is known to underestimate barrier heights.^48^

To improve our estimate of the reaction profile, we have implemented a correction to the simulated data using calculations at higher levels of theory (see the Methods section). Our final best estimate for the activation barrier and reaction energy lies in a range of 21.8 ± 1.2 and −1.4 ± 0.8 kcal/mol, respectively. Thus, we predict the reaction to be thermodynamically allowed, but only marginally so.

Our barrier estimation is markedly lower than that by Kikuchi et al. (28.7 kcal/mol), who used an implicit solvation model of water to study the same nucleophilic attack but in the presence of ammonium and hydronium cations.^40^ Of course, the preference for forming ion pairs in highly polar media will depend on the ion concentration. However, given that our modeling results predict a lower barrier and because the concentration of ammonia (or other bases) used in typical HCN polymerization experiments tends to be low (0.03–0.5 M),^18,49^ we can conclude that (a) cation coordination is not necessary for iminoacetonitrile formation and that (b) active involvement of solvent molecules (and explicit consideration of these interactions in modeling) is necessary to explain the base catalysis of this reaction.

### Implications of Our Results for the Polymerization of HCN

Polymerization of HCN typically results in complex reaction mixtures with many high-molecular-weight products that have poor solubility in common solvents.^50^ Product compositions of such experiments also vary significantly with reaction conditions such as initial concentrations,^51^ the presence of oxygen, and the degree of conversion.^52^ These often ill-understood products may play important roles in astro- and prebiotic chemistry. As a consequence, definite identification and characterization of products of HCN polymerization are both important and exceedingly challenging. Here, we aim to better understand the potential role of iminoacetonitrile in the formation of HCN polymers. We do so by comparing our theoretical predictions of iminoacetonitrile formation kinetics with observed reaction rates in HCN polymerization experiments. Of course, the rate of HCN dimerization is not necessarily the same as that of its polymerization or processes leading to other reaction products. Implicit in our following comparison with the experiment are two assumptions: first, that the initial step in HCN’s base-catalyzed polymerization proceeds through the formation of iminoacetonitrile, and, second, that this initial step is rate-determining. Aside from its known rapid polymerization into a “red-brown-black material” above 233 K,^38^ the precise reactivity and scope of iminoacetonitrile chemistry are largely unknown and will be the subject of future work.

**Figure 4:**
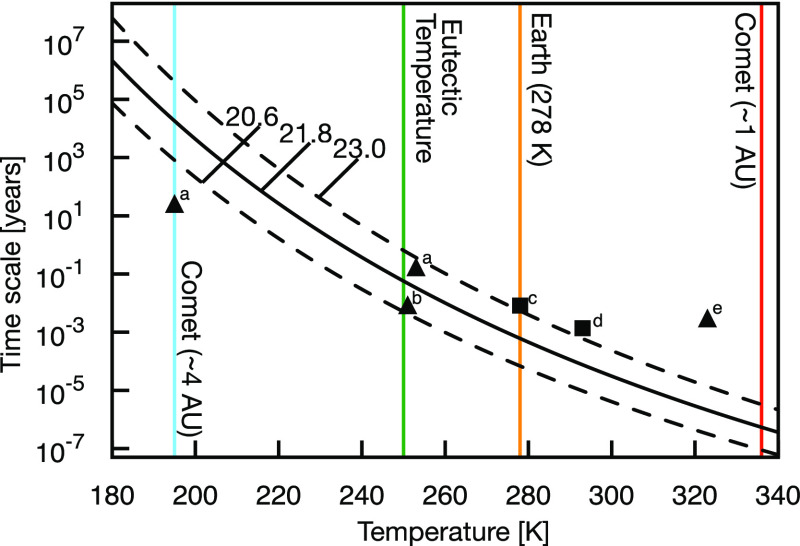
Predicted and experimental time scales of HCN polymerization. Iminoacetonitrile formation (black solid line) estimated using the Eyring equation and an assumed pseudo-first-order reaction rate. Dashed lines are upper and lower bounds resulting from statistical uncertainty in our simulations at 278 K. Assuming pseudo-zero-order rate kinetics would result in a time scale of the same order of magnitude in pure HCN but would be less accurate for aqueous solutions of HCN. Reported experimental time scales are shown for neat HCN polymerization (squares) and in aqueous solution (triangles) and are indicated as ^a^Levy et al.,^34^^b^Sanchez et al.,^2^^c^Mamajanov and Herzfeld,^18^^d^He et al.,^19^ and ^e^Mas et al.^53^ Colored vertical lines indicate the temperature in our simulations (orange), representative measures of cometary surfaces in the inner solar system (blue and red),^54^ and the eutectic freezing temperature of HCN and water mixtures (green).^55^

In Figure 4, we have assumed pseudo-first-order kinetics for iminoacetonitrile formation and extrapolated the corresponding reaction time scales to temperatures that are both higher and lower than our simulation temperature of 278 K. We stress that the extrapolation of our results to temperatures other than 278 K comes with various approximations, the first one being negligible changes to the height of the Gibbs reaction barrier with temperature. Our extrapolation also implies that mass transfer limitations remain similar to those in the liquid state and that the reaction mechanism does not change. It is, consequently, expected that our estimates may disagree with observed time scales of reactions in low-temperature (<200 K) and high-temperature (>300 K) conditions. Shown together with our data in Figure 4 are experimental time scales for HCN polymerization reported for several different conditions.

Close to a simulation temperature of 278 K, a barrier of 22 kcal/mol should proceed on the order of days (Figure 4). In other words, our rate estimate of condensed phase HCN dimerization is in reasonable agreement with the time scales of pure HCN polymerization observed by Mamajanov and Herzfeld at 278 K^18^ and He et al.^19^ at room temperature. The temperature dependence of the tetramerization rate measured at 283–313 K by Sanchez et al.^17^ suggests an apparent activation barrier of 20.5 kcal/mol, also in close agreement with our barrier estimate. Our predicted reaction time scale also agrees well with aqueous HCN polymerization experiments performed by Miller and colleagues^34^ as well as Sanchez et al.^2^ near 250 K. We here remind that aqueous HCN solutions that are close to or below the eutectic point (250 K) form a eutectic phase in which HCN is concentrated (to 74.5 mole percent).^55^ We can speculate that effects associated with such a phase may be one reason for the sometimes strikingly different experimental results near the eutectic temperature. For example, whereas Sanchez et al. have reported the formation of the HCN tetramer diaminomaleonitrile after days at 251 K,^2^ Marin-Yaseli et al. were unable to observe meaningful reactivity after a month of reaction time at 253 K.^49^

As expected, our approximate extrapolation in Figure 4 does not agree as well with experimental measurements performed at low temperatures. In particular, the one-of-a-kind experiment by Miller and colleagues, in which polymerization of HCN at 195 K was observed over ∼25 years,^34^ implies a lower reaction barrier in the largely frozen solution compared to that predicted for the liquid (albeit due to a low ammonia–water eutectic temperature of ∼173 K, the aqueous NH_4_CN solution may not have been entirely frozen^34^). The latter comparison implies that crystal surfaces and defects in frozen HCN solutions may be better polymerization catalysts compared to the liquid system studied herein. Relatedly, reaction kinetics studies performed between 323 and 363 K by Mas et al. suggest that different reaction mechanisms, and barrier heights, are at play at higher temperatures.^53^

### Possible Role of Iminoacetonitrile in Different Astrochemical Environments

The time scale required for the studied dimerization drops rapidly with temperature (c.f., Figure 4), with important consequences for the possible environments in which the reaction mechanism may be relevant. Our simulations correspond well to setups of some laboratory experiments, but one might question the direct relevance of these conditions in nature. Where might there exist, or have existed, concentrated solutions of HCN? The atmospheric production rate of HCN on the early Earth has been predicted to be 10^–8^–10^–6^ cm^2^/s and much of what was produced is believed to have been deposited on Earth’s surface (30 million tons/year).^14^ However, despite a substantial production in the atmosphere, the concentration of HCN in Earth’s early oceans is likely to have been much too low to allow for polymerization.^56^ Eutectic freezing could have occurred in shallow pools or under very harsh glacier conditions and corresponds to a plausible route to concentrated aqueous HCN.^17,23^ Our calculations predict that the 250 K eutectic temperature of a HCN–water mixture should allow for the formation of iminoacetonitrile, and possibly subsequent HCN polymerization, over a time scale of months.

Observations of cometary coma^4^ have established comets as another environment rich in HCN, and the possibility of HCN polymerization in comets has been pointed out by many (see, e.g., refs (57, 58)). The surface temperature of comets varies considerably with their proximity to a star (from ∼190 K at 4 AU to ∼340 K at 1 AU).^54^ Heating-and-cooling cycles of comets may allow the formation of eutectic solutions of HCN with a high enough concentration to allow for iminoacetonitrile formation on relevant time scales. We stress that the formation of such solutions requires pressures of hundreds of Pa, an order of magnitude higher than that expected on the surface of comets. Whether subsurface liquids can form during heating of the cometary surface, or during cometary explosive eruptions, is not known and is the subject of ongoing research.^54^

HCN is one of the main products of the atmospheric photochemistry on Saturn’s moon Titan.^8^ The temperature of Titan’s atmosphere varies drastically with altitude but ranges from 200 K at 300 km^59^ to ∼94 K at the surface.^60^ The formed HCN can be expected to deposit on the surface and in the hydrocarbon seas of Titan in large amounts.^10,61^ The abundance of HCN dissolved in the hydrocarbon seas is uncertain but predicted to be low^62,63^ and orders of magnitudes smaller than the lowest reported molar concentration used in aqueous HCN polymerization experiments (0.02 mole %).^17^ The cyanide anion, the catalyst in our study, is believed to be the most common anion in the chemical haze surrounding the large moon.^64^ However, provided that we assume a similar reaction barrier in the hydrocarbon seas of Titan as in neat HCN, the low surface temperature (<94 K^65^) would hinder the reaction from occurring within relevant time scales. Whereas solid-state equivalences to the reaction considered herein might prove operable over very long time scales, our current simulations describe HCN in the liquid state. In other words, for the iminoacetonitrile formation reaction considered in this work to be feasible on Titan, temporary heating of HCN surface deposits or melting of atmospheric aerosols would be required. Such heating might be generated by impact events or, possibly, coincide with runaway exothermic polymerization of acetylene, another main product of Titan’s atmospheric chemistry.^66−68^

## Conclusions

HCN in the liquid state is complex, featuring an extremely large dielectric constant, strong directional intermolecular interactions, and acid–base equilibria that allows for its participation in catalytic cycles. Our simulation of base-catalyzed dimerization of HCN explains the formation of iminoacetonitrile in the liquid state. The time scales estimated for iminoacetonitrile formation are similar to those observed for HCN polymerization experiments, which suggests that the dimer formation may be the rate-limiting step for a host of subsequent reaction chemistry, including polymerization. The predicted rate of formation of iminoacetonitrile allows for its formation in several environments and supports a potential central role of the molecule in astro- and prebiotic chemistry. Barring detection by nuclear magnetic resonance and vibrational spectroscopy or successful trapping experiments, we suggest that the laser desorption ionization of frozen reaction mixtures coupled to time-of-flight mass spectroscopy may provide one route to experientially proving the role of iminoacetonitrile in HCN polymerization.

## Methods

### Molecular Dynamics

Ab initio molecular dynamics simulations were performed with CP2K v6.1^69^ at the Perdew–Burke–Ernzerhof (PBE)^70^/DZVP–Goedecker–Teter–Hutter (GTH)^71^ level of theory, including the D3 dispersion correction by Grimme et al.^72^ The presence of anions and hydrogen bonds in the simulations merits the inclusion of diffuse functions in the basis set. However, such functions affect relative energies by 1.1 kcal/mol or less (Supporting Information Table 1) and were omitted in the interest of reducing computational costs. The GTH pseudopotential^73^ was used with a cutoff of 280 Rydberg. Liquid HCN was simulated in a cubic box with a side length of 15.94 Å subjected to periodic boundary conditions. The number of molecules were tailored to 64, including one cyanide anion, so as to accurately reproduce the density of HCN at 278 K (0.709 g/cm^3^).^74^ The system was propagated in an NVT ensemble at 278 K with a 0.5 fs time step. The steered simulations and the subsequent committor analysis simulations were performed with the canonical sampling through velocity rescaling thermostat^75^ and a thermostat time constant of 50 fs.

### Metadynamics

The *s* and *z* path-collective variables were constructed following the example of Branduardi et al.^43^ (see Supporting Information Section 4). The path-collective variables used during the metadynamics^76^ simulations were defined based on the bond topology of the carbon in the cyanide anion, through the distance function by Pietrucci and Saitta.^77^ The PLUMED library version 2.5.0^78^ was used to steer the dynamics simulations. Two reference structures were used: a cyanide dissolved in HCN and a solvated iminoacetonitrile, leaving the path between those two states open for exploration. Following a 5 ps equilibration, the reference structures were simulated for 20 ps prior to an analysis of their coordination patterns. The metadynamics simulation was initiated from the reactant state.

### Committor Analysis and Umbrella Sampling

The transition state (TS) of the reaction observed in the metadynamics simulation was identified through a committor analysis.^79^ Ten out of 20 trajectories starting from the TS structure ended up in the reactant basin and in the product basin, respectively. A free-energy profile of the reaction was then constructed using umbrella sampling^80^ along the *s* coordinate using configurations from trajectories obtained in the committor analysis as starting points. The umbrella sampling simulations were run for 11 ps after 1.5 ps of equilibration. The force constants, *k*, used for the harmonic potentials in these windows (Supporting Information Table 2) were chosen so that , where σ^2^ is the variance of the *s* coordinate in a typical simulation. The umbrella sampling windows were spaced at most 0.025 s coordinate units apart (corresponding to 3% of the distance between the *s* coordinates of reactants and products). Together with the chosen force constants, this separation provided a good overlap of the sampling in different windows (Supporting Information Figure 2). The weighted histogram analysis method (WHAM)^81^ version 2.0.9 was used to retrieve the free-energy profile of the reaction. Uncertainties in the energy estimates were estimated using a block averaging method in WHAM.^82^

### Energy Correction to the Free-Energy Profile

As a necessary compromise between accuracy and computational cost, all simulations were done using the PBE-D3 functional. The largest contribution to the error in the free-energy profile can be attributed to inherent errors in the potential used to calculate the electronic (Born–Oppenheimer) energy Δ*E*. This conclusion was reached by comparing the thermal corrections Δ*G* – Δ*E* when calculated with PBE-D3 and the more accurate B3LYP^83,84^-D3 method (Supporting Information Section 1). Hybrid exchange–correlation functionals, such as B3LYP, represent the most accurate level of theory that can be practically implemented on our system. The B3LYP functional has reported average errors of 2 and 4 kcal/mol for association reactions and hydrogen transfer, respectively.^48^

To reduce overall errors in the free energy, an electronic correction term, Δ*E*_corr_, was added to the free-energy profile Δ*G*_PBE_, according to Δ*G*_final_ = Δ*G*_PBE_ + Δ*E*_corr_, where Δ*G*_final_ is our best estimate of the relative Gibbs energy (see Figure S4). The correction term, Δ*E*_corr_, was obtained by averaging 105 energy evaluations of configurations from the umbrella sampling windows corresponding to reactant (*s* value 1.09), transition state (*s* value 1.32), and product (*s* value 1.88) at two levels of theory. The configurations were taken at 0.1–0.2 ps intervals from the trajectories. Δ*E*_corr_ was calculated as Δ*E*_corr_ = Δ*E*_B3LYP-D3,avg_ – Δ*E*_PBE-D3,avg_, where the latter two terms are average energy differences calculated with B3LYP-D3 and PBE-D3, respectively. The Δ*E*_corr_ calculations were performed with the Vienna ab initio simulation package (VASP) version 5.4.4.^85^ Standard projected-augmented wave potentials^86^ were used together with a plane-wave energy cutoff of 800 eV and a convergence criterion of 10^–4^ meV/atom. All VASP calculations were done at the Γ point only.

## Supporting Information Available

Link to database with molecular structures and simulation inputs; comparison of functionals; benchmark of basis sets; parameters used in umbrella sampling simulations; figure with histograms from umbrella sampling simulations; figure of the one-dimensional free-energy profile and statistical error bars; detailed description of parameters used in metadynamics simulations, path-collective variables and parameters used to construct them; figure of free-energy profile before and after applying the energy correction scheme; and relative energy of the iminoacetonitrile isomers (PDF)

## Abbreviations Used

HCN: hydrogen cyanide
DFT: density functional theory
PBE: Perdew–Burke–Ernzerhof
VASP: Vienna ab initio simulation package
B3LYP: Becke, 3-parameter, Lee–Yang–Parr

## References

[ref1] Oró J. Mechanism of synthesis of adenine from HCN under possible primitive earth conditions. Nature 1961, 191, 1193–1194. 10.1038/1911193a0. 13731264

[ref2] Sanchez R.; Ferris J.; Orgel L. E. Conditions for Purine Synthesis: Did Prebiotic Synthesis Occur at Low Temperatures. Science 1966, 153, 72 10.1126/science.153.3731.72. 5938419

[ref3] Buhl D.; Snyder L. E. Unidentified Interstellar Microwave Line. Nature 1970, 228, 267 10.1038/228267a0. 16058492

[ref4] Rodgers S. D.; Charnley S. B. HNC and HCN in Comets. Astrophys. J., Lett. 1998, 501, L227 and references therein 10.1086/311459.

[ref5] Tokunaga A. T.; Beck S. C.; Geballe T. R.; Lacy J. H.; Serabyn E. The detection of HCN on Jupiter. Icarus 1981, 48, 283–289. 10.1016/0019-1035(81)90109-3.

[ref6] Marten A.; et al. First Observations of CO and HCN on Neptune and Uranus at Millimeter Wavelengths and Their Implications for Atmospheric Chemistry. Astrophys. J. 1993, 406, 285 10.1086/172440.

[ref7] Lellouch E.; et al. Detection of CO and HCN in Pluto’s atmosphere with ALMA. Icarus 2017, 286, 289–307. 10.1016/j.icarus.2016.10.013.

[ref8] Molter E. M.; et al. Alma observations of HCN and its isotopologues on titan. Astron. J. 2016, 152, 42 and references therein 10.3847/0004-6256/152/2/42.

[ref9] Sagan C.; Khare B. N. Tholins: organic chemistry of interstellar grains and gas. Nature 1979, 277, 102–107. 10.1038/277102a0.

[ref10] Anderson C. M.; Samuelson R. E.; Nna-Mvondo D. Organic Ices in Titan’s Stratosphere. Space Sci. Rev. 2018, 214, 125 10.1007/s11214-018-0559-5.

[ref11] Sutherland J. D. The Origin of Life-Out of the Blue. Angew. Chem. Int. Ed. 2016, 55, 104–121. 10.1002/anie.201506585. 26510485

[ref12] Teanby N. A.; et al. Vertical profiles of HCN, HC3N, and C2H2 in Titan’s atmosphere derived from Cassini/CIRS data. Icarus 2007, 186, 364–384. 10.1016/j.icarus.2006.09.024.

[ref13] Rahm M.; Lunine J. I.; Usher D.; Shalloway D. Polymorphism and electronic structure of polyimine and its potential significance for prebiotic chemistry on Titan. Proc. Natl. Acad. Sci. U.S.A. 2016, 113, 8121–8126. 10.1073/pnas.1606634113. 27382167 PMC4961190

[ref14] Tian F.; Kasting J. F.; Zahnle K. Revisiting HCN formation in Earth’s early atmosphere. Earth Planet. Sci. Lett. 2011, 308, 417–423. 10.1016/j.epsl.2011.06.011.

[ref15] Ferus M.; et al. High Energy Radical Chemistry Formation of HCN-rich Atmospheres on early Earth. Sci. Rep. 2017, 7, 6275 10.1038/s41598-017-06489-1. 28740207 PMC5524942

[ref16] Airapetian V. S.; Glocer A.; Gronoff G.; Hébrard E.; Danchi W. Prebiotic chemistry and atmospheric warming of early Earth by an active young Sun. Nat. Geosci. 2016, 9, 452–455. 10.1038/ngeo2719.

[ref17] Sanchez R. A.; Ferris J. P.; Orgel L. E. Prebiotic synthesis. II. Synthesis of purine precursors and amino acids from aqueous hydrogen cyanide. J. Mol. Biol. 1967, 30, 223–253. 4297187

[ref18] Mamajanov I.; Herzfeld J. HCN polymers characterized by solid state NMR: Chains and sheets formed in the neat liquid. J. Chem. Phys. 2009, 130, 134503 10.1063/1.3092908. 19355747 PMC2832022

[ref19] He C.; Lin G.; Upton K. T.; Imanaka H.; Smith M. A. Structural Investigation of HCN Polymer Isotopomers by Solution-State Multidimensional NMR. J. Phys. Chem. A 2012, 116, 4751–4759. 10.1021/jp301604f. 22530720

[ref20] Völker T. Polymere Blausäure. Angew. Chem., Int. Ed. 1960, 72, 379–384. 10.1002/ange.19600721104.

[ref21] Moffat J. B. Three dimers of hydrogen cyanide: iminoacetonitrile, aminocyanocarbene, and azacyclopropenylidenimine; geometry-optimized Ab initio energies. J. Chem. Soc., Chem. Commun. 1975, 888–890. 10.1039/c39750000888.

[ref22] Ruiz-Bermejo M.; de la Fuente J. L.; Pérez-Fernández C.; Mateo-Martí E. A Comprehensive Review of HCN-Derived Polymers. Processes 2021, 9, 597 and references therein 10.3390/pr9040597.

[ref23] Ruiz-Bermejo M.; Zorzano M.-P.; Osuna-Esteban S. Simple organics and biomonomers identified in HCN polymers: an overview. Life 2013, 3, 421–448. 10.3390/life3030421. 25369814 PMC4187177

[ref24] Miller S. L. A Production of Amino Acids under Possible Primitive Earth. Science 1953, 117, 528–529. 10.1126/science.117.3046.528. 13056598

[ref25] Marín-Yaseli M. R.; Mompeán C.; Ruiz-Bermejo M. A Prebiotic Synthesis of Pterins. Chem.-Eur. J. 2015, 21, 13531–13534. 10.1002/chem.201502345. 26256284

[ref26] Rivilla V. M.; et al. Abundant Z-cyanomethanimine in the interstellar medium: paving the way to the synthesis of adenine. Mon. Not. R. Astron. Soc.: Lett. 2019, 483, L114–L119. 10.1093/mnrasl/sly228.

[ref27] Zaleski D. P.; et al. Detection of E-Cyanomethanimine Toward Sagittarius B2(N) in the Green Bank Telescope Primos Survey. Astrophys. J. 2013, 765, L10 10.1088/2041-8205/765/1/L10.

[ref28] Yim M. K.; Choe J. C. Dimerization of HCN in the gas phase: A theoretical mechanistic study. Chem. Phys. Lett. 2012, 538, 24–28. 10.1016/j.cplett.2012.04.042.

[ref29] Benallou A. Understanding the most favourable dimer of HCN for the oligomerization process in the gas phase of interstellar clouds. Comput. Theor. Chem. 2016, 1097, 79–82. 10.1016/j.comptc.2016.10.016.

[ref30] Nandi S.; Bhattacharyya D.; Anoop A. Prebiotic Chemistry of HCN Tetramerization by Automated Reaction Search. Chem.-Eur. J. 2018, 24, 4885–4894. 10.1002/chem.201705492. 29369429

[ref31] Moffat J. B.; Tang K. F. A theoretical study of the reactive dimerization of HCN. J. Theor. Biol. 1976, 58, 83–95. 10.1016/0022-5193(76)90140-5. 183061

[ref32] Choe J. C. Dimerization of HCN in Interstellar Icy Grain Mantles: A DFT Study. Bull. Korean Chem. Soc. 2019, 40, 205–206. 10.1002/bkcs.11666.

[ref33] Zhang X.; et al. Chemical models of interstellar cyanomethanimine isomers. Mon. Not. R. Astron. Soc. 2020, 497, 609–625. 10.1093/mnras/staa1979.

[ref34] Levy M.; Miller S. L.; Brinton K.; Bada J. L. Prebiotic Synthesis of Adenine and Amino Acids Under Europa-like Conditions. Icarus 2000, 145, 609–613. 10.1006/icar.2000.6365. 11543508

[ref35] Villafañe-Barajas S. A.; Ruiz-Bermejo M.; Rayo-Pizarroso P.; Colín-García M. Characterization of HCN-Derived Thermal Polymer: Implications for Chemical Evolution. Processes 2020, 8, 968 10.3390/pr8080968.

[ref36] Ferris J. P.; Donner D. B.; Lotz W. Chemical evolution. IX. Mechanism of the oligomerization of hydrogen cyanide and its possible role in the origins of life. J. Am. Chem. Soc. 1972, 94, 6968–6974. 10.1021/ja00775a018. 5072331

[ref37] Lorencak P.; Raabe G.; Radziszewski J. J.; Wentrup C. Iminoacetonitrile, a hydrogen cyanide dimer; IR identification in an argon matrix. J. Chem. Soc., Chem. Commun. 1986, 916–918. 10.1039/c39860000916.

[ref38] Evans R. A.; Lorencak P.; Ha T. K.; Wentrup C. HCN dimers: iminoacetonitrile and N-cyanomethanimine. J. Am. Chem. Soc. 1991, 113, 7261–7276. 10.1021/ja00019a026.

[ref39] Evans R. A.; Lacombe S. M.; Simon M. J.; Pfister-Guillouzo G.; Wentrup C. Hydrogen cyanide dimers: photoelectron spectrum of iminoacetonitrile. J. Phys. Chem. A 1992, 96, 4801–4804. 10.1021/j100191a015.

[ref40] Kikuchi O.; Watanabe T.; Satoh Y.; Inadomi Y. Ab initio GB study of prebiotic synthesis of purine precursors from aqueous hydrogen cyanide: dimerization reaction of HCN in aqueous solution. J. Mol. Struct.: THEOCHEM 2000, 507, 53–62. 10.1016/S0166-1280(99)00356-5.

[ref41] Coates G. E.; Coates J. E. Hydrogen Cyanide. Part XIII. The Dielectric Constant of Anhydrous Hydrogen Cyanide. J. Chem. Soc. 1944, 77–81. 10.1039/jr9440000077.

[ref42] Hobbs M. E.; Jhon M. S.; Eyring H. The dielectric constant of liquid water and various forms of ice according to significant structure theory. Proc. Natl. Acad. Sci. U.S.A. 1966, 56, 31–38. 10.1073/pnas.56.1.31. 16591362 PMC285670

[ref43] Branduardi D.; Gervasio F. L.; Parrinello M. From A to B in free energy space. J. Chem. Phys. 2007, 126, 054103 10.1063/1.2432340. 17302470

[ref44] Martiniano H. F. M. C.; Costa Cabral B. J. Structure and electronic properties of a strong dipolar liquid: Born-Oppenheimer molecular dynamics of liquid hydrogen cyanide. Chem. Phys. Lett. 2013, 555, 119–124. 10.1016/j.cplett.2012.10.080.

[ref45] Jung S. H.; Choe J. C. Mechanisms of Prebiotic Adenine Synthesis from HCN by Oligomerization in the Gas Phase. Astrobiology 2013, 13, 465–475. 10.1089/ast.2013.0973. 23659646

[ref46] Smith I. W. M.; Talbi D.; Herbst E. The production of HCN dimer and more complex oligomers in dense interstellar clouds. Astron. Astrophys. 2001, 369, 611–615. 10.1051/0004-6361:20010126.

[ref47] Mandal S.; Nair N. N. Speeding-up ab initio molecular dynamics with hybrid functionals using adaptively compressed exchange operator based multiple timestepping. J. Chem. Phys. 2019, 151, 151102 10.1063/1.5125422. 31640357

[ref48] Zhao Y.; Truhlar D. G. Design of Density Functionals That Are Broadly Accurate for Thermochemistry, Thermochemical Kinetics, and Nonbonded Interactions. J. Phys. Chem. A 2005, 109, 5656–5667. 10.1021/jp050536c. 16833898

[ref49] Marin-Yaseli M. R.; Moreno M.; Briones C.; de L. F. J. L.; Ruiz-Bermejo M. Experimental conditions affecting the kinetics of aqueous HCN polymerization as revealed by UV–vis spectroscopy. Spectrochim. Acta, Part A 2018, 191, 389–397. 10.1016/j.saa.2017.10.003. 29065330

[ref50] Marín-Yaseli M. R.; Cid C.; Yagüe A. I.; Ruiz-Bermejo M. Detection of Macromolecular Fractions in HCN Polymers Using Electrophoretic and Ultrafiltration Techniques. Chem. Biodiversity 2017, 14, e1600241 10.1002/cbdv.201600241. 27518115

[ref51] Ruiz-Bermejo M.; et al. A Comparative Study on HCN Polymers Synthesized by Polymerization of NH4 CN or Diaminomaleonitrile in Aqueous Media: New Perspectives for Prebiotic Chemistry and Materials Science. Chem. Eur. J. 2019, 25, 11437–11455. 10.1002/chem.201901911. 31373416

[ref52] Fernández A.; Ruiz-Bermejo M.; de la Fuente J. L. Modelling the kinetics and structural property evolution of a versatile reaction: aqueous HCN polymerization. Phys. Chem. Chem. Phys. 2018, 20, 17353–17366. 10.1039/C8CP01662C. 29905340

[ref53] Mas I.; de la Fuente J. L.; Ruiz-Bermejo M. Temperature effect on aqueous NH4CN polymerization: Relationship between kinetic behaviour and structural properties. Eur. Polym. J. 2020, 132, 109719 10.1016/j.eurpolymj.2020.109719.

[ref54] Suttle M. D.; Folco L.; Genge M. J.; Russell S. S. Flying too close to the Sun – The viability of perihelion-induced aqueous alteration on periodic comets. Icarus 2020, 351, 113956 10.1016/j.icarus.2020.113956.

[ref55] Coates J. E.; Harthorne N. H. Studies on hydrogen cyanide. Part III. The Freezing Points of Hydrogen Cyanide. J. Chem. Soc. 1931, 0, 657–665. 10.1039/JR9310000657.

[ref56] Miyakawa S.; Cleaves H. J.; Miller S. L. The Cold Origin of Life: B. Implications Based on Pyrimidines and Purines Produced From Frozen Ammonium Cyanide Solutions. Orig. Life Evol. Biosph. 2002, 32, 209–218. 10.1023/A:1019514022822. 12227425

[ref57] Rettig T. W.; Tegler S. C.; Pasto D. J.; Mumma M. J. Comet Outbursts and Polymers of HCN. Astrophys. J. 1992, 398, 293 10.1086/171857.

[ref58] Matthews C. N.; Minard R. D. Hydrogen cyanide polymers, comets and the origin of life. Faraday Discuss. 2006, 133, 393–401. 10.1039/b516791d. 17191459

[ref59] Mathé C.; et al. Seasonal changes in the middle atmosphere of Titan from Cassini/CIRS observations: Temperature and trace species abundance profiles from 2004 to 2017. Icarus 2020, 344, 113547 10.1016/j.icarus.2019.113547.

[ref60] Cottini V.; et al. Spatial and temporal variations in Titan’s surface temperatures from Cassini CIRS observations. Planet. Space Sci. 2012, 60, 62–71. 10.1016/j.pss.2011.03.015.

[ref61] Mastrogiuseppe M.; et al. The bathymetry of a Titan sea. Geophys. Res. Lett. 2014, 41, 1432–1437. 10.1002/2013GL058618.

[ref62] Stevenson J. M.; et al. Solvation of nitrogen compounds in Titan’s seas, precipitates, and atmosphere. Icarus 2015, 256, 1–12. 10.1016/j.icarus.2015.04.019.

[ref63] Cordier D.; Mousis O.; Lunine J. I.; Lavvas P.; Vuitton V. Erratum: “An estimate of the chemical composition of Titan’s lakes”. Astrophys. J. 2013, 768, L23 10.1088/2041-8205/768/1/l23.

[ref64] Millar T. J.; Walsh C.; Field T. A. Negative Ions in Space. Chem. Rev. 2017, 117, 1765–1795. 10.1021/acs.chemrev.6b00480. 28112897

[ref65] Jennings D. E.; et al. Titan Surface Temperatures during the Cassini Mission. Astrophys. J. 2019, 877, L8 10.3847/2041-8213/ab1f91.

[ref66] Artemieva N.; Lunine J. I. Impact cratering on Titan II. Global melt, escaping ejecta, and aqueous alteration of surface organics. Icarus 2005, 175, 522–533. 10.1016/j.icarus.2004.12.005.

[ref67] Lunine J. I.; Cable M. L.; Glein C. R.; Hörst S. M.; Rahm M. In Planetary Astrobiology, Meadows V.; Arney G. N.; Schmidt B. E.; Des Marais D. J., Eds.; University of Arizona: Tuscon, 2020; pp 247–266.

[ref68] Nixon C. A.; et al. Titan’s cold case files - Outstanding questions after Cassini-Huygens. Planet. Space Sci. 2018, 155, 50–72. 10.1016/j.pss.2018.02.009.

[ref69] Hutter J.; Iannuzzi M.; Schiffmann F.; VandeVondele J. CP2K: atomistic simulations of condensed matter systems. Wiley Interdiscip. Rev. Comput. Mol. Sci. 2014, 4, 15–25. 10.1002/wcms.1159.

[ref70] Perdew J. P.; Burke K.; Ernzerhof M. Generalized gradient approximation made simple. Phys. Rev. Lett. 1996, 77, 3865–3868. 10.1103/PhysRevLett.77.3865. 10062328

[ref71] VandeVondele J.; Hutter J. Gaussian basis sets for accurate calculations on molecular systems in gas and condensed phases. J. Chem. Phys. 2007, 127, 114105 10.1063/1.2770708. 17887826

[ref72] Grimme S.; Ehrlich S.; Goerigk L. Effect of the damping function in dispersion corrected density functional theory. J. Comput. Chem. 2011, 32, 1456–1465. 10.1002/jcc.21759. 21370243

[ref73] Goedecker S.; Teter M.; Hutter J. Separable dual-space Gaussian pseudopotentials. Phys. Rev. B 1996, 54, 1703–1710. 10.1103/PhysRevB.54.1703. 9986014

[ref74] Coates J. E.; Davies R. H. 246. Studies on hydrogen cyanide. Part XVIII. Some physical properties of anhydrous hydrogen cyanide. J. Chem. Soc. 1950, 1194–1199. 10.1039/jr9500001194.

[ref75] Bussi G.; Donadio D.; Parrinello M. Canonical sampling through velocity rescaling. J. Chem. Phys. 2007, 126, 014101 10.1063/1.2408420. 17212484

[ref76] Barducci A.; Bonomi M.; Parrinello M. Metadynamics. WIREs Comput. Mol. Sci. 2011, 1, 826–843. 10.1002/wcms.31.

[ref77] Pietrucci F.; Saitta A. M. Formamide reaction network in gas phase and solution via a unified theoretical approach: Toward a reconciliation of different prebiotic scenarios. Proc. Natl. Acad. Sci. U.S.A. 2015, 112, 15030 10.1073/pnas.1512486112. 26598679 PMC4679036

[ref78] Tribello G. A.; Bonomi M.; Branduardi D.; Camilloni C.; Bussi G. PLUMED 2: New feathers for an old bird. Comput. Phys. Commun. 2014, 185, 604–613. 10.1016/j.cpc.2013.09.018.

[ref79] Bolhuis P. G.; Chandler D.; Dellago C.; Geissler P. L. Transition path sampling: Throwing ropes over rough mountain passes, in the dark. Annu. Rev. Phys. Chem. 2002, 53, 291–318. 10.1146/annurev.physchem.53.082301.113146. 11972010

[ref80] Torrie G. M.; Valleau J. P. Nonphysical sampling distributions in Monte Carlo free-energy estimation: Umbrella sampling. J. Comput. Phys. 1977, 23, 187–199. 10.1016/0021-9991(77)90121-8.

[ref81] Grossfield A.. WHAM: the Weighted Histogram Analysis Method. Version 2.0.9. http://membrane.urmc.rochester.edu/wordpress/?page_id=126.

[ref82] Flyvbjerg H. In Advances in Computer Simulation, Kertész J.; Kondor I., Eds.; Springer Berlin Heidelberg: Berlin, Heidelberg, 1998; pp 88–103.

[ref83] Becke A. D. A new mixing of Hartree-Fock and local density-functional theories. J. Chem. Phys. 1993, 98, 1372–1377. 10.1063/1.464304.

[ref84] Stephens P. J.; Devlin F. J.; Chabalowski C. F.; Frisch M. J. Ab Initio Calculation of Vibrational Absorption and Circular Dichroism Spectra Using Density Functional Force Fields. J. Phys. Chem. A 1994, 98, 11623–11627. 10.1021/j100096a001.

[ref85] Kresse G.; Furthmueller J. Efficient iterative schemes for ab initio total-energy calculations using a plane-wave basis set. Phys. Rev. B 1996, 54, 11169–11186. 10.1103/PhysRevB.54.11169. 9984901

[ref86] Blöchl P. E. Projector augmented-wave method. Phys. Rev. B 1994, 50, 17953–17979. 10.1103/PhysRevB.50.17953. 9976227

