# Supplementary material for: The Beginning of HCN Polymerization: Iminoacetonitrile Formation and Its Implications in Astrochemical Environments
Source: ACS Earth Space Chem. 2021 Jul 29;5(8):2152–9. doi: 10.1021/acsearthspacechem.1c00195 (PMC8397470; doi:10.1021/acsearthspacechem.1c00195)
Supplement: Supplementary file 1 — sp1c00195_si_001.pdf [file sp1c00195_si_001.pdf]

Supplementary Information for

The Beginning of HCN Polymerization: Iminoacetonitrile formation and Its Implications in Astrochemical Environments

Hilda Sandström[1]. Martin Rahm[1]*.
[1]Department of Chemistry and Chemical Engineering, Chalmers University of Technology, Gothenburg, Sweden SE-412 96.

*Martin Rahm
Email: martin.rahm@chalmers.se

This PDF file includes:

- Supplementary text
- Figures 1 to 5
- Tables 1 to 4
- SI References

Structures and simulation inputs are freely available at the Swedish National Data Service (SND): https://snd.gu.se/en/catalogue/study/2021-131

And can be cited as:
Hilda Sandström, Martin Rahm. Chalmers University of Technology, Chemistry and Chemical Engineering (2021). *Theoretical studies of iminoacetonitrile formation*. Swedish National Data Service. Version 1. https://doi.org/10.5878/37fw-8a25

1. Comparison of functionals using implicit solvation modeling

Figure 1 shows the relative energy profile of iminoacetonitrile formation in the liquid state computed with an implicit solvation model. An implicit treatment of the surrounding chemical environment allows for a comparison between the PBE [1] functional with a double-ζ basis set, which was used in our molecular dynamics simulations, with the more accurate hybrid functional B3LYP[2,3] with a larger triple-ζ basis set. Both profiles in Fig. 1 include the Grimme D3 dispersion corrections[4]. The molecular optimizations were performed in Gaussian 16, revision B.01[5,6], using its well-tested default polarizable continuum model (PCM)[6]. We employed the standard PCM parameters of water, a molecule with a similar size as HCN, but modified the dielectric constant to that of HCN (85.8 → 144.8)[7]. Computations with PBE-D3 relied on the 6-31G(d,p) basis set, which is of comparable size to the DZVP-Goedecker-Teter-Hutter[8] (GTH) used for simulations with CP2K[9] (DZVP-GTH is unavailable in Gaussian16). Computations with B3LYP-D3 were made with the large 6-311++G(d,p) basis set.

Figure 1 shows the electronic energy profile and the Gibbs free energy profile of iminoacetonitrile formation computed with the implicit solvation model. There is a noticeable difference in the reaction barrier (5.2 kcal/mol) and reaction energy (3.7 kcal/mol) of the two electronic energy profiles. The thermal corrections calculated for the transition state and product are -0.6 and +4.0 for PBE-D3, respectively, and +2.3 and +5.5 for B3LYP-D3, respectively.

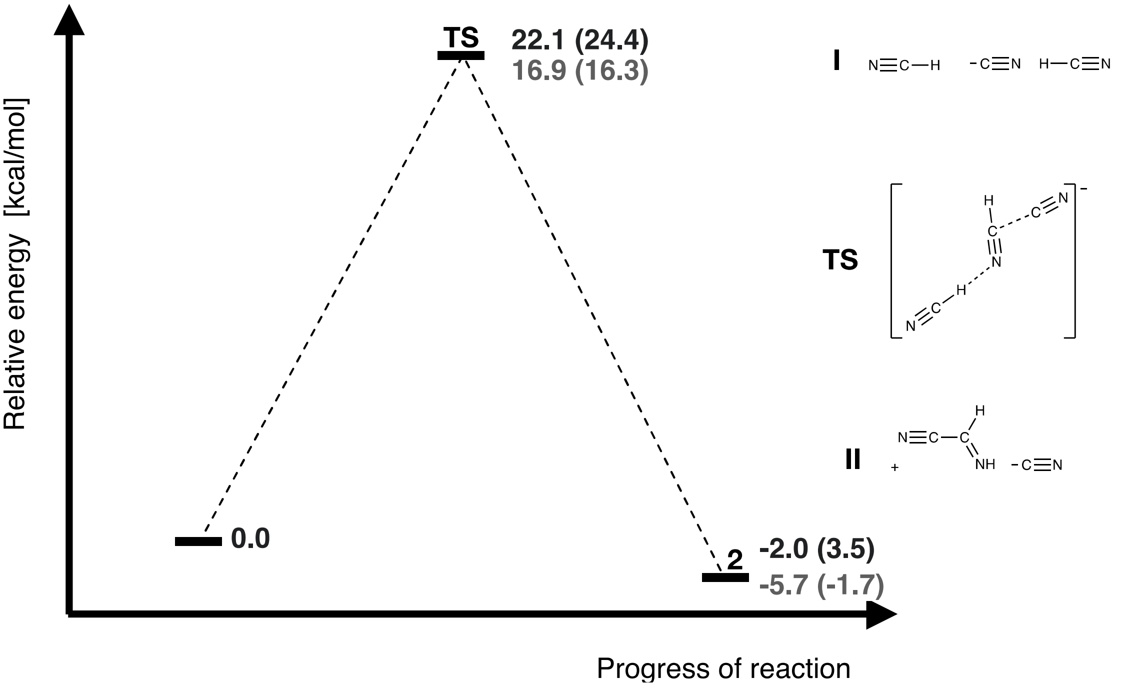

Fig. 1. Relative reaction energy (Δ*E*) profile for iminoacetonitrile formation modeled in implicit HCN solvent. The corresponding Gibbs energy profile (ΔG^0) is shown in parenthesis. Black values correspond to B3LYP-D3/6-311++G(d,p) and grey values to PBE-D3/6-31G(d,p).

2. The effect of basis sets for simulations

Molecular dynamics is computationally expensive, requiring the evaluation of energies and forces for all ~10^5 time steps of a typical simulation. A small basis set can drastically increase the computational speed. On the other hand, the error caused by using a too small basis set affects the quality of the energies, and in turn also the dynamics. For example, diffuse functions can be important for a good description of highly coordinated and charged systems, such as cyanide anions and HCN. In Table 1, we compare relative energies computed using a double-ζ DZVP-GTH basis set with (aug-) and without diffuse functions in CP2K[9]. Table 1 shows the computed gas phase hydrogen bond energy of the HCN dimer, and the gas phase reaction barrier and reaction energy for base-catalyzed iminoacetonitrile formation. Because the effects of the diffuse functions are small (<1.1 kcal/mol) (Table 1), the smaller DZVP-GTH basis set was chosen for all dynamics simulations of the liquid state.

Table 1. Reaction energy and barrier for iminoacetonitrile formation and HCN hydrogen bond energy in gas phase, calculated with and without diffuse basis functions.

Method/ basis set	Hydrogen bond strength[a] [kcal/mol]	Reaction barrier[b] [kcal/mol]	Reaction energy[b] [kcal/mol]
PBE-D3/aug-DZVP-GTH	5.7	22.1	1.0
PBE-D3/DZVP-GTH	6.2	21.0	1.7

[a] Computed for two HCN molecules. [b] Energies are computed for geometries optimized at the PCM-B3LYP-D3/6-311++G(d,p) level of theory (see Figure 1).

The method that we use lacks a fully quantum mechanical treatment of the nuclear dynamics, including. e.g., tunneling. Such effects are known to play a role in the hydrogen bonding capabilities of water (see e.g., Ref (10)). More sophisticated methodology, such as ring-polymer molecular dynamics would be needed to incorporate such effects but are prohibitively expensive in our case.

3. Umbrella sampling

Table 2. Parameters used to describe the harmonic potentials in umbrella sampling windows. One force constant is given for each center (*s* coordinate) parameter. The *s* coordinate denotes the position of the simulated system along the reaction path. The smallest value (1.09) indicates the position of the reactants and the largest value (1.88) indicates the position of the products. The standard deviation of the *s* coordinate, σ, was 0.002 in the simulation of the reactant state and 0.01 for the product state.

s coordinate	Force constant kJ/mol	s value	Force constant kJ/mol
1.091966	12000	1.503721	9000
1.100168	12000	1.538	15000
1.125519	12000	1.551113	9000
1.149964	12000	1.582307	9000
1.175036	12000	1.604077	9000
1.201518	12000	1.632824	9000
1.222563	12000	1.652299	9000
1.249984	12000	1.677486	9000
1.265625	12000	1.703093	9000
1.291652	15000	1.726402	9000
1.300306	12000	1.749995	9000
1.300306	15000	1.777263	9000
1.323677	15000	1.800887	12000
1.353149	12000	1.800887	15000
1.382975	15000	1.800887	9000
1.405368	15000	1.82485	9000
1.419272	15000	1.851294	9000
1.447349	15000	1.880939	9000
1.483562	15000		

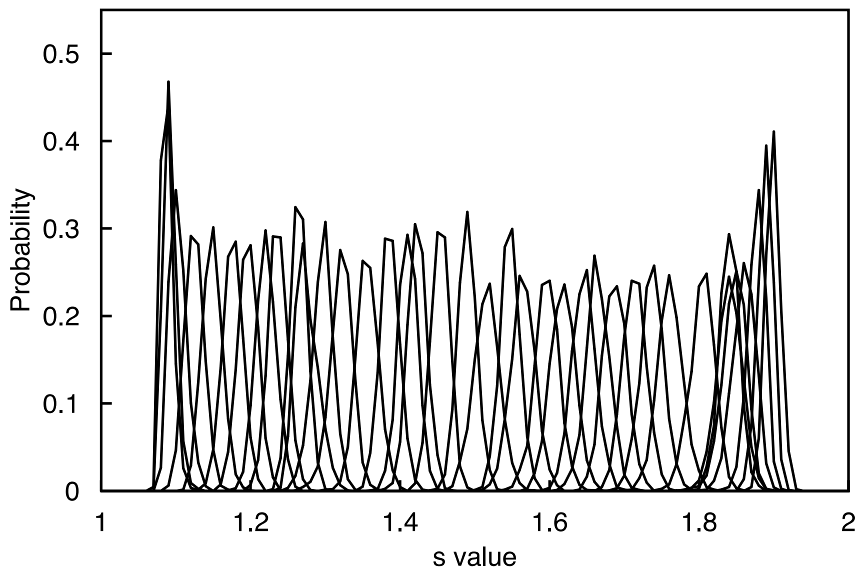

Fig. 2. Histograms showing the distributions of the *s* coordinate in each of the umbrella sampling windows. The large overlap between the distributions increases the quality of the free energy profile computed with the weighted histogram analysis method (WHAM).

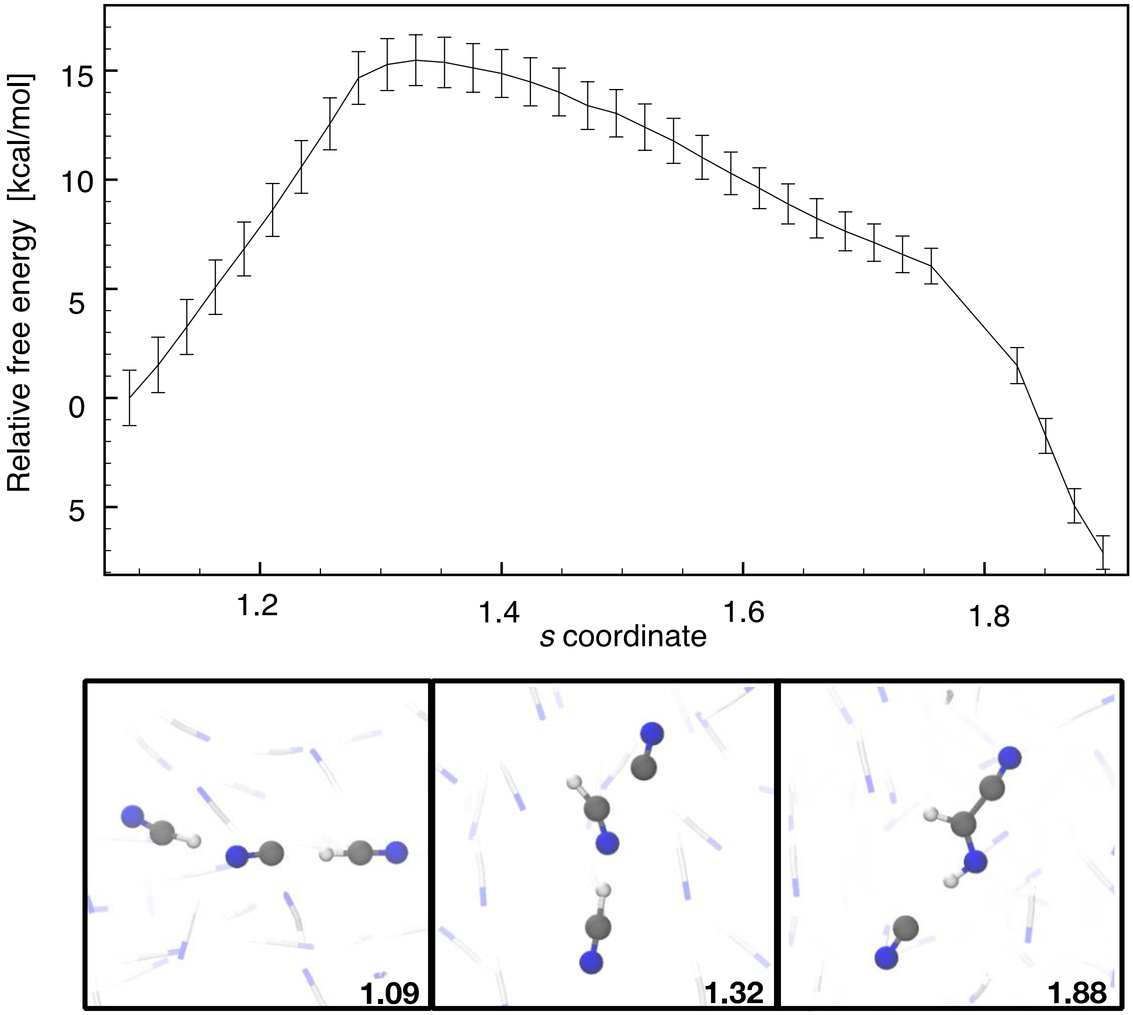

Fig. 3. Top: Free energy profile of dimerization obtained using umbrella sampling at the PBE-D3/DZVP-GTH level of theory. The error bars were obtained using block averaging and correspond to standard deviations. Bottom: Representative structure at three different *s* coordinates corresponding to reactant (1.09), transition state (1.32), and products (1.88).

4. Parameters for path collective variables and metadynamics

Our path collective variables depend on a distance function $D_k(t)$ and a parameter called λ according to $s(t) = \frac{\sum_{k=1}^{N_k} k e^{-\lambda D_k(t)}}{\sum_{k'=1}^{N_k} e^{-\lambda D_k(t)}}$ and $z(t) = -\frac{1}{\lambda} log\left(\sum_{k=1}^{N_k} e^{-\lambda D_k(t)}\right)$ where k is the reference structure index and N_k is the number of reference structures. The path collective variables, *s* and *z*, used during the metadynamics simulations were defined based on the bond topology of the carbon in the cyanide anion. This dependence is described by the distance function by Pietrucci and Saitta, $D_k(t) = \sum_{IS}[C_{IS}(t) - C_{IS}^k]^2$ (10), where $C_{IS}(t)$ is the coordination between atom *I* and atoms of type *S* at time *t*, and C_{IS}^k is the corresponding coordination of the atom in reference structure k. The coordination is calculated according to $C_{IS}(t) = \sum_{J \in S} \frac{\left[1-\left(\frac{R_{IJ}(t)}{R_{SS'}^0}\right)^N\right]}{\left[1-\left(\frac{R_{IJ}(t)}{R_{SS'}^0}\right)^M\right]}$ where R_{IJ}(t) is the Euclidean distance between atoms *I* and *J*, and R_{SS}^0 is a cutoff that is used as a reference distance, specific for atom types *S* and *S'*. *N* and *M* are parameters that determine how sharp the switching function is. We have set *N* to 8 and *M* to 16 following recommendations of Pérez-Villa et al. [11]. The reference cutoff distance, $R_{SS'}^0$, between groups of carbon and nitrogen atoms was set to of 1.8 Å, and to 1.5 Å for distances to hydrogen atoms.

The reference states were simulated with a Nose-Hoover thermostat of length 4 and a time constant of 500 fs[12]. The canonical sampling through velocity rescaling was a better fit for the steered simulations[13]. The GTH-DZVP basis set and a GTH pseudopotentials were used with energy cutoff of 200 Rydberg[8].

The specified coordination patterns of the cyanide anion and HCN in the reactant state are listed in Table 3, and for the atoms of iminoacetonitrile in the product state in Table 4. In the metadynamics simulation, we based the path collective variables on the coordination of the carbon of the cyanide anion only. Excluding the coordination of a specific HCN makes all the solvent HCN molecules indistinguishable substrates for the cyanide anion. We also excluded the coordination to hydrogen atoms during the metadynamics simulation so that the *s*- and *z* coordinates would be unaffected by potential proton transfers that otherwise would change the topology. The coordination used in the metadynamics simulations are marked in bold in Tables 3 and 4.

Table 3. Coordination patterns, $\boldsymbol{C_{IS}^R}$, of atoms in the cyanide anion (denoted by subscript a), and HCN in the reactant state. A value close to 1 indicates a covalent bond.

	C	N	H
C_a	**0.04**	0.98	0.16
N_a	1.01	0.01	0.22
C	0.02	1.00	0.93
N	1.00	0.02	0.11

Table 4. Coordination patterns C_{IS}^{P} of carbon and nitrogen atoms in iminoacetonitrile in the product state. A value close to 1 indicates a covalent bond.

	C	N	H
C_a	**0.85**	1.08	0.06
N_a	1.04	0.02	0.06
C	0.85	1.00	1.04
N	1.07	0.03	1.09

In addition to the coordination pattens, both the s- and z coordinates depend on the parameter λ, according to the description in Computational Methods of the main text. The λ parameter was chosen such that the reference frames were distanced 0.8 s coordinate units apart, which ensures a smooth behavior of the s and z variables[11]. λ was set to 3.47 for the explorative metadynamics based on the difference between the coordination patterns in Tables 3 and 4. For the subsequent committor analysis and umbrella sampling, the coordination of all atoms in Tables 3 and 4 were used to obtain a better description of the reaction (including the proton transfer). λ was then set to 0.97. The width of the deposited potential was set to 0.05 and 0.01 in the s and z coordinate, respectively. A deposition rate of 100 fs was used together with a bias of 5 kJ/mol.

5. Energy correction to the free energy profile

The energy corrections computed with the Vienna ab initio simulation package (VASP, version 5.4.4)[14] are +6.3 kcal/mol for the transition state and +5.7 kcal/mol for the product (Fig. 4). The standard deviation of the electronic energy in the different umbrella sampling windows is < 0.1 kcal mol^{-1} atom^{-1}. The energy fluctuations arise mainly due to the dynamic solvent environment. As the fluctuations are of similar size in all windows, error cancellation is expected to occur when calculating the average relative energy of the different structures.

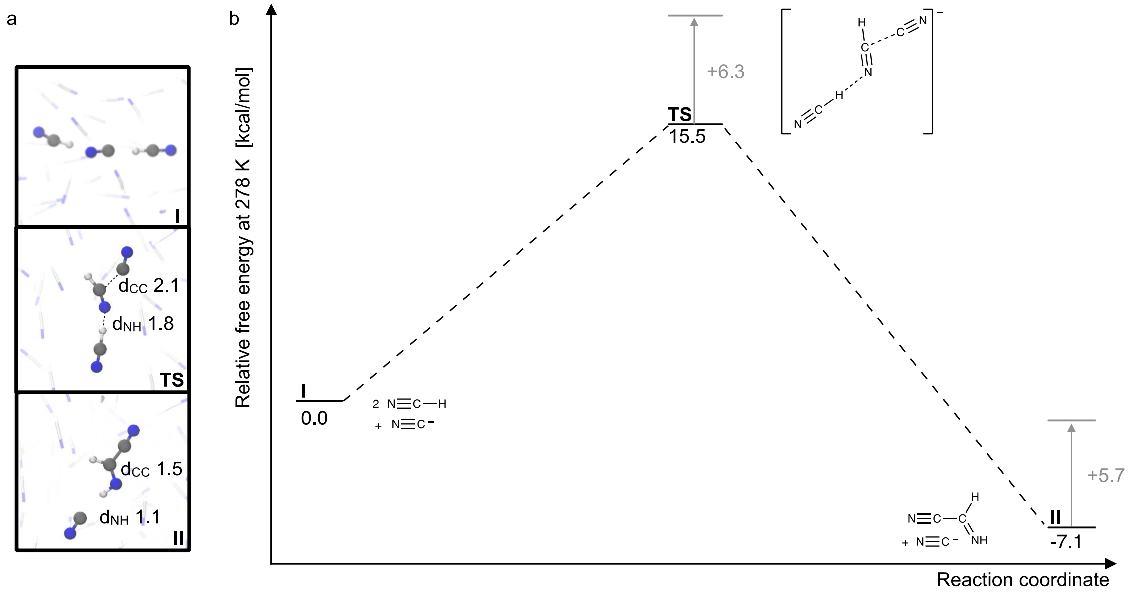

Fig. 4. Panel a. Representative configurations of the reactant (**I**), transition state (**TS**) and product (**II**) in the molecular dynamics simulations. **Panel b.** Free energy profile of iminoacetonitrile formation at 278 K. Values in black correspond to the relative energies obtained from the umbrella sampling simulations. Values in grey correspond to the energy correction computed as described in the Methods section of the main text.

6. Relative energy of iminoacetonitrile isomers

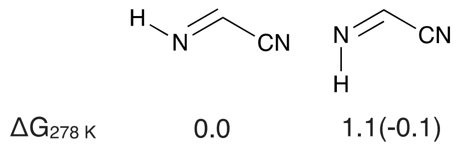

Fig. 5. The relative energy of the *Z* and *E*-iminoacetonitrile isomers computed with an implicit solvent model. Corresponding values in vacuum are shown within parenthesis. Energies were computed at the CCSD(T)[15]/6-311++G(d,p)//B3LYP-D3/6-311++G(d,p) level of theory. The *E*-form is predicted to be more stable in a polar solvent environment whereas the *Z*-form is more stable in vacuum.

7. Full reference for Gaussian16 Rev B01

Frisch, M. J.; Trucks, G. W.; Schlegel, H. B.; Scuseria, G. E.; Robb, M. A.; Cheeseman, J. R.; Scalmani, G.; Barone, V.; Petersson, G. A.; Nakatsuji, H.; Li, X.; Caricato, M.; Marenich, A. V.; Bloino, J.; Janesko, B. G.; Gomperts, R.; Mennucci, B.; Hratchian, H. P.; Ortiz, J. V.; Izmaylov, A. F.; Sonnenberg, J. L.; Williams-Young, D.; Ding, F.; Lipparini, F.; Egidi, F.; Goings, J.; Peng, B.; Petrone, A.; Henderson, T.; Ranasinghe, D.; Zakrzewski, V. G.; Gao, J.; Rega, N.; Zheng, G.; Liang, W.; Hada, M.; Ehara, M.; Toyota, K.; Fukuda, R.; Hasegawa, J.; Ishida, M.; Nakajima, T.; Honda, Y.; Kitao, O.; Nakai, H.; Vreven, T.; Throssell, K.; Montgomery, J. A., Jr.; Peralta, J. E.; Ogliaro, F.; Bearpark, M. J.; Heyd, J. J.; Brothers, E. N.; Kudin, K. N.; Staroverov, V. N.; Keith, T. A.; Kobayashi, R.; Normand, J.; Raghavachari, K.; Rendell, A. P.; Burant, J. C.; Iyengar, S. S.; Tomasi, J.; Cossi, M.; Millam, J. M.; Klene, M.; Adamo, C.; Cammi, R.; Ochterski, J. W.; Martin, R. L.; Morokuma, K.; Farkas, O.; Foresman, J. B.; Fox, D. J. Gaussian 16, Revision B.01. Gaussian, Inc., Wallingford CT,; 2016

SI references

(1) Perdew, J. P.; Burke, K.; Ernzerhof, M. Generalized gradient approximation made simple. *Phys. Rev. Lett.* **1996,** *77*, 3865-3868.

(2) Becke, A. D. A new mixing of Hartree-Fock and local density-functional theories. *J. Chem. Phys.* **1993,** *98*, 1372-1377.

(3) Stephens, P. J.; Devlin, F. J.; Chabalowski, C. F.; Frisch, M. J. Ab Initio Calculation of Vibrational Absorption and Circular Dichroism Spectra Using Density Functional Force Fields. *J. Phys. Chem.* **1994,** *98*, 11623-11627.

(4) Grimme, S.; Ehrlich, S.; Goerigk, L. Effect of the damping function in dispersion corrected density functional theory. *J. Comput. Chem.* **2011,** *32*, 1456-1465.

(5) Frisch, M. J. et al. *Gaussian 16, Revision B.01*, Gaussian, Inc., Wallingford CT, 2016.

(6) Tomasi, J.; Mennucci, B.; Cammi, R. Quantum Mechanical Continuum Solvation Models. *Chem. Rev.* **2005,** *105*, 2999-3093.

(7) Coates, G. E.; Coates, J. E. Hydrogen Cyanide. Part XIII. The Dielectric Constant of Anhydrous Hydrogen Cyanide. *J Chem Soc* **1944,** 77-81.

(8) VandeVondele, J.; Hutter, J. Gaussian basis sets for accurate calculations on molecular systems in gas and condensed phases. *J. Chem. Phys.* **2007,** *127*, 114105.

(9) Hutter, J.; Iannuzzi, M.; Schiffmann, F.; VandeVondele, J. CP2K: atomistic simulations of condensed matter systems. *Wiley Interdiscip. Rev. Comput. Mol. Sci.* **2014,** *4*, 15-25.

(10) Markland, T. E.; Ceriotti, M. Nuclear quantum effects enter the mainstream. *Nat Rev Chem* **2018,** *2*, 0109.

(11) Pérez-Villa, A. et al. Synthesis of RNA Nucleotides in Plausible Prebiotic Conditions from ab Initio Computer Simulations. *J. Phys. Chem. Lett.* **2018,** *9*, 4981-4987.

(12) Evans, D. J.; Holian, B. L. The Nose–Hoover thermostat.s*J. Chem. Phys.* **1985,** *83*, 4069-4074.

(13) Bussi, G.; Donadio, D.; Parrinello, M. Canonical sampling through velocity rescaling. *J. Chem. Phys.* **2007,** *126*, 014101.

(14) Kresse, G.; Furthmueller, J. Efficient iterative schemes for ab initio total-energy calculations using a plane-wave basis set. *Phys. Rev. B: Condens. Matter* **1996,** *54*, 11169-11186.

(15) Purvis, G. D., III; Bartlett, R. J. A full coupled-cluster singles and doubles model: the inclusion of disconnected triples. *J. Chem. Phys.* **1982,** *76*, 1910-1918.
